# Idiopathic Granulomatous Mastitis in a Postmenopausal Woman Mimicking Breast Carcinoma: A Diagnostic Challenge

**DOI:** 10.7759/cureus.106027

**Published:** 2026-03-28

**Authors:** Georgia Xanthopoulou, Evangelos Solakis, Christos Barkolias, Kyriaki Theodorolea, Ilektra Spyrou, Nikolaos P Tasis

**Affiliations:** 1 Department of Surgery, Naval and Veterans Hospital of Athens, Athens, GRC; 2 Department of Surgery, 401 Military Hospital of Athens, Athens, GRC

**Keywords:** breast cancer, granulomatous lobular mastitis, idiopathic granulomatous mastitis (igm), inflammatory diseases of the breast, lobular mastitis

## Abstract

Idiopathic granulomatous mastitis is a rare benign inflammatory condition of the breast characterized by granulomatous inflammation affecting the lobular units. Clinically, it most commonly presents as a palpable breast mass with pain, erythema, or skin thickening, and may be accompanied by abscess formation or fistula development. Because these findings overlap significantly with those of breast carcinoma, the disease frequently poses a diagnostic challenge for clinicians. We report a case of idiopathic granulomatous mastitis in a postmenopausal, 55-year-old patient presenting with extensive inflammatory breast disease and imaging findings highly suspicious for malignancy. Core needle biopsy was negative, showing a discrepancy with the highly suspicious imaging and clinical characteristics, which led to operative management with lumpectomy.

## Introduction

Idiopathic granulomatous mastitis (IGM), also known as granulomatous lobular mastitis, is a rare benign inflammatory condition of the breast, characterized by granulomatous inflammation affecting the lobular units, that can mimic infection or malignancy. Although first described in 1972, its pathogenesis remains incompletely understood. Proposed etiologic mechanisms include autoimmune reactions, infectious agents, hormonal influences, and localized inflammatory responses to extravasated ductal secretions [1-4].

Clinically, granulomatous mastitis most commonly presents as a palpable breast mass associated with pain, erythema, or skin thickening and may be accompanied by abscess formation or fistula development. Because these findings overlap significantly with those of breast carcinoma, the disease frequently poses a diagnostic challenge for clinicians and radiologists [1,3]. The disease usually affects women of reproductive age, and presentation in postmenopausal women is relatively uncommon and may further increase suspicion for malignancy [5,6].

We report a case of IGM in a postmenopausal patient presenting with extensive inflammatory breast disease and imaging findings highly suspicious for malignancy, which led to operative management. The case highlights the diagnostic difficulties associated with this condition and emphasizes the importance of integrating clinical, radiologic, and histopathologic findings when managing suspicious breast conditions.

## Case presentation

A 55-year-old, nulliparous, postmenopausal woman presented to our breast department with a painful retroareolar mass in the right breast associated with progressive inflammatory changes. Her medical history was unremarkable, with no regular medication use reported and no known drug allergies. She was an active smoker. Family history was notable for ovarian cancer in her maternal aunt and breast cancer in the daughter of the same aunt.

The patient initially reported localized breast pain, erythema, and swelling in the retroareolar region. Empirical antibiotic therapy was administered for 14 days for presumed infectious mastitis; however, no clinical improvement was observed. On physical examination, a tender retroareolar mass was palpable in the right breast with associated inflammatory signs.

**Figure 1:**
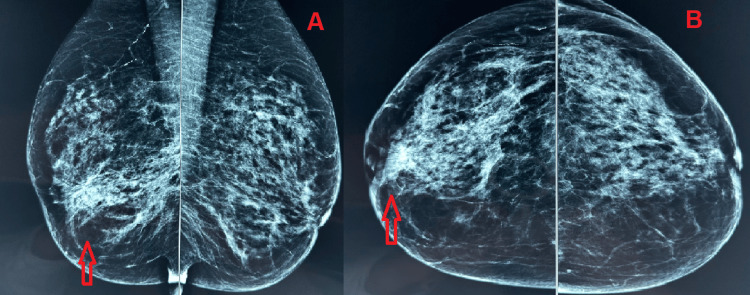
Breast mammography with red arrow pointing to the lesion A. Mediolateral oblique; B. craniocaudal.

Breast ultrasonography demonstrated an extensive heterogeneous area within the right breast, predominantly involving the lower outer quadrant, accompanied by retraction of the ipsilateral nipple. Duct ectasia of the lactiferous ducts and increased vascularity were also noted. No pathologically enlarged axillary lymph nodes were identified. Mammography revealed dense breasts with a micronodular parenchymal pattern. New findings included focal skin thickening, retraction of the right nipple, and nodular enhancement measuring approximately 2 × 2.5 cm in the retroareolar region of the right breast, located approximately 1.5 cm from the nipple (Figure 1). The lesion was classified as BI-RADS 5 [7] in both imaging modalities.

**Figure 2:**
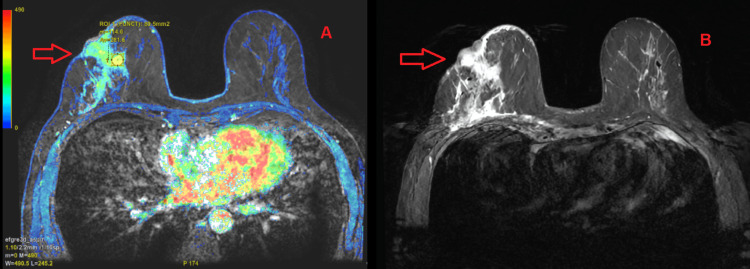
Breast magnetic resonance imaging with red arrow pointing to the lesion A. T2-weighted processed dynamic sequence with parametric enhancement map showing early enhancement within the region of interest. B. T2-weighted fat-suppressed sequence demonstrating the enhancing lesion for anatomical correlation.

Given the highly suspicious imaging findings, an ultrasound-guided core needle biopsy was performed; however, histopathologic evaluation was negative for malignancy. Because of the discrepancy between imaging and pathology, breast magnetic resonance imaging (MRI) was subsequently performed. MRI demonstrated an extensive pathological area in the right breast originating in the retroareolar region and extending posteriorly toward the anterior chest wall, measuring approximately 7.4 cm in maximum diameter (Figure 2). The lesion was also classified as BI-RADS 5 [7].

**Figure 3:**
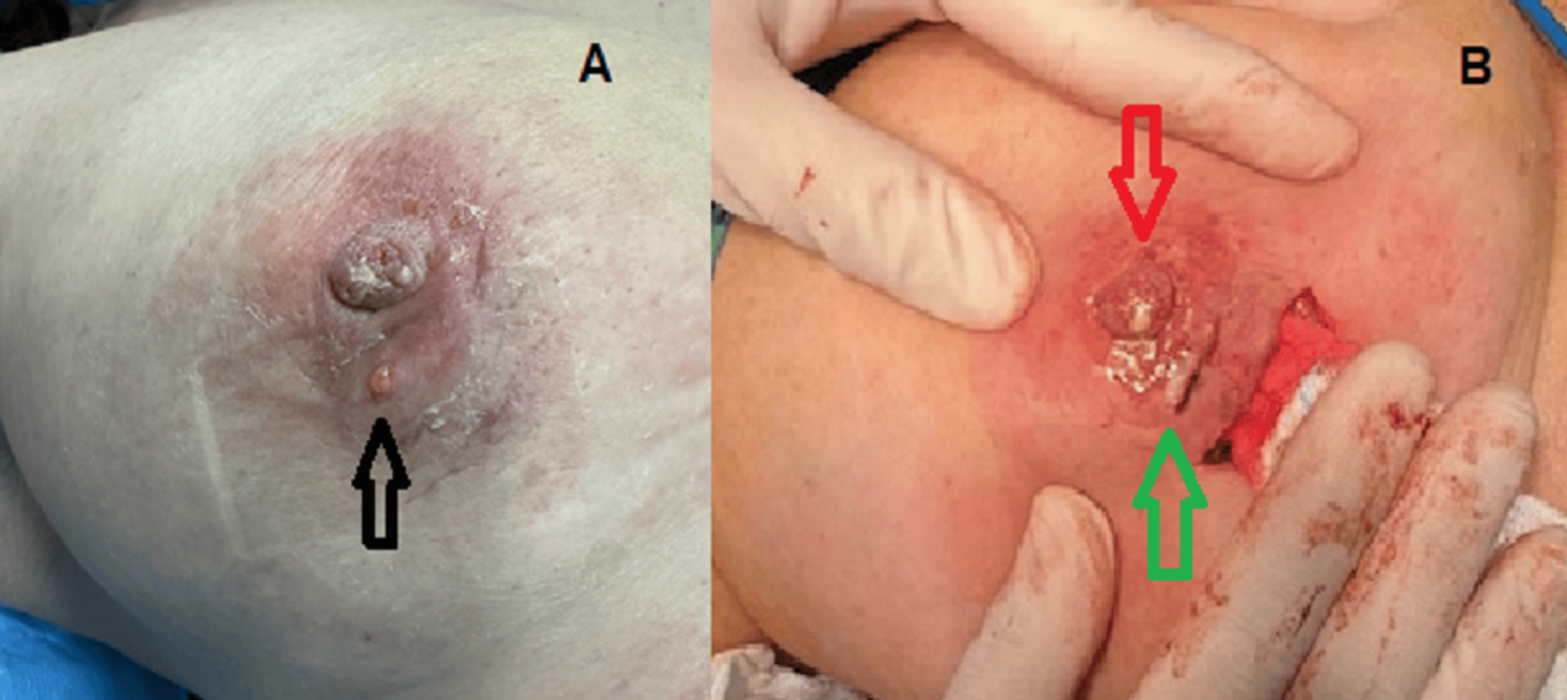
Preoperative (A) and intraoperative (B) nipple and periareolar findings Black arrow: Preoperative periareolar fistula. Red arrow: Pus oozing from nipple intraoperatively. Green arrow: Incision for the intraoperative full-thickness areolar frozen section, which came up negative.

Despite ongoing antibiotic treatment, the patient’s clinical condition progressively deteriorated, with worsening inflammatory changes and the development of multiple cutaneous fistulas (Figure 3).

Due to the persistent high clinical and radiologic suspicion for malignancy despite negative biopsy findings, the multidisciplinary team (MDT) proposed surgical management.

During surgery, full-thickness frozen-section analysis of the areolar region and subareolar mass took place with no evidence of malignancy (Figure 3). Purulent material was observed oozing from the nipple intraoperatively (Figure 3). Given the extensive inflammatory mass and the discordance between imaging and biopsy results, the decision was made to proceed with excision of the mass together with the nipple-areolar complex. In addition, polymerase chain reaction (PCR) testing for *Mycobacterium tuberculosis* was requested to exclude tuberculous mastitis, which came up negative.

Gross pathological examination revealed a mass measuring approximately 5 cm in maximum diameter. Microscopic examination demonstrated extensive inflammatory involvement of the breast parenchyma characterized by a dense mixed inflammatory infiltrate composed predominantly of lymphocytes and plasma cells, with the presence of neutrophils forming areas of abscess formation. Numerous epithelioid histiocytes and multinucleated giant cells were also identified, with strong CD68 positivity on immunohistochemistry. Immunohistochemical staining demonstrated residual damaged glandular structures within the inflamed lobules with preservation of the myoepithelial cell layer (CKAE1/AE3-positive epithelial cells and peripheral CK14-positive myoepithelial cells), confirming the non-neoplastic nature of the lesion. The overall histologic and immunophenotypic findings were consistent with granulomatous perilobular mastitis with associated abscess formation, and no evidence of malignancy.

The patient’s postoperative course was uneventful, with progressive resolution of the inflammatory process. At four-year follow-up, she remained asymptomatic with no evidence of recurrence.

## Discussion

Granulomatous mastitis is a rare inflammatory breast disease that often mimics breast carcinoma both clinically and radiologically, frequently leading to a diagnostic challenge [1,3,5]. Because imaging findings may closely resemble those of malignancy, histopathologic evaluation remains essential for establishing a definitive diagnosis [3].

The clinical manifestations include those of local breast inflammation. A systematic review analyzing more than 3000 patients reported that a palpable mass occurs in approximately 80% of cases, while pain is present in nearly two-thirds of patients [4]. The disease typically affects women of reproductive age and is strongly associated with recent pregnancy or lactation [4,8]. Postmenopausal presentation, as observed in our patient, is uncommon and may further complicate diagnostic assessment because malignancy is usually more prevalent in this age group.

Radiologic evaluation plays an important role in the initial assessment of breast lesions; however, imaging findings in granulomatous mastitis are frequently nonspecific. Mammography may demonstrate asymmetric densities, parenchymal distortion, or skin thickening, while ultrasonography often reveals irregular hypoechoic masses with increased vascularity [1]. MRI may show extensive non-mass enhancement or inflammatory infiltration that may be indistinguishable from invasive carcinoma [5,9].

In our case, the lesion was classified as BI-RADS 5 [7] across all imaging modalities, representing one of the strongest possible radiologic suspicions for malignancy. Although core needle biopsy is indicated for evaluating suspicious breast lesions, in heterogeneous inflammatory processes such as granulomatous mastitis, results are often equivocal. Persistent discordance between imaging findings and histopathology should therefore prompt further investigation.

The optimal management of granulomatous mastitis remains controversial, and no standardized therapeutic algorithm has been universally accepted. Reported treatment strategies include observation, antibiotics, corticosteroid therapy, immunosuppressive agents such as methotrexate, and surgical excision [4,10,11]. Corticosteroids are considered the most commonly used medical treatment because of the presumed autoimmune and inflammatory pathogenesis of the disease. In a systematic review of 3060 patients, corticosteroid therapy was administered in approximately 69% of cases, antibiotics in about 55%, and surgical treatment in approximately 65% [4].

Systemic corticosteroids have been reported to achieve clinical remission in a substantial proportion of patients and are often considered first-line therapy for moderate or extensive disease. However, steroid therapy may be associated with adverse effects and relatively high recurrence rates after treatment discontinuation [10]. In recent years, immunosuppressive agents such as methotrexate have been added as steroid-sparing therapies, particularly in patients with recurrent and refractory disease, or those who are unable to tolerate prolonged corticosteroid treatment [11,12].

Surgical management remains an important option, particularly in patients with localized disease, persistent abscess formation, or fistulizing lesions that fail to respond to medical therapy. Surgical procedures range from wide local excision to more extensive resections, depending on the extent of disease. Although surgery may achieve definitive resolution in many patients, recurrence has been reported, especially when excision margins are inadequate or when surgery is performed during the active inflammatory phase [11,13]. Consequently, current consensus recommendations emphasize an individualized treatment strategy based on disease severity, extent of inflammation, and patient factors [11].

In the present case, the progressive inflammatory course, fistula formation, and discordant imaging prompted surgical treatment despite biopsy results. Surgical excision resulted in complete resolution of the disease, and no recurrence has been observed during four years of follow-up.

## Conclusions

Granulomatous mastitis is a rare benign inflammatory breast disease that may closely mimic breast carcinoma both clinically and radiologically. Histopathologic examination remains essential for accurate diagnosis. Persistent discordance between imaging findings and biopsy results should prompt multidisciplinary evaluation and, when necessary, surgical exploration. Awareness of this condition is important to avoid misdiagnosis and ensure appropriate management of suspicious breast lesions.
